# Vagal neuron expression of the microbiota-derived metabolite receptor, free fatty acid receptor (FFAR3), is necessary for normal feeding behavior

**DOI:** 10.1016/j.molmet.2021.101350

**Published:** 2021-10-06

**Authors:** Tyler M. Cook, Chaitanya K. Gavini, Jason Jesse, Gregory Aubert, Emily Gornick, Raiza Bonomo, Laurent Gautron, Brian T. Layden, Virginie Mansuy-Aubert

**Affiliations:** 1 Department of Cell and Molecular Physiology, Stritch School of Medicine, Loyola University Chicago, Maywood IL, 60153, USA; 2 Department of Internal Medicine, Division of Cardiology, Stritch School of Medicine, Loyola University Chicago, Maywood, IL 60153, USA; 3 Center for Hypothalamic Research, Department of Internal Medicine, The University of Texas Southwestern Medical Center, 5323 Harry Hines Blvd., Dallas, 75390, TX, USA; 4 Division of Endocrinology, Diabetes and Metabolism, Department of Medicine, University of Illinois at Chicago, Chicago, IL, USA

**Keywords:** Short chain fatty acid receptor, Vagal neuron, Gut microbiome, Food intake, Gut-brain axis, Satiety, Microbiota, Fiber, Obesity

## Abstract

**Objective:**

The vagus nerve provides a direct line of communication between the gut and the brain for proper regulation of energy balance and glucose homeostasis. Short-chain fatty acids (SCFAs) produced *via* gut microbiota fermentation of dietary fiber have been proposed to regulate host metabolism and feeding behavior *via* the vagus nerve, but the molecular mechanisms have not yet been elucidated. We sought to identify the G-protein-coupled receptors within vagal neurons that mediate the physiological and therapeutic benefits of SCFAs.

**Methods:**

SCFA, particularly propionate, signaling occurs *via* free fatty acid receptor 3 (FFAR3), that we found expressed in vagal sensory neurons innervating throughout the gut. The lack of cell-specific animal models has impeded our understanding of gut/brain communication; therefore, we generated a mouse model for cre-recombinase-driven deletion of *Ffar3*. We comprehensively characterized the feeding behavior of control and vagal-FFAR3 knockout (KO) mice in response to various conditions including fasting/refeeding, western diet (WD) feeding, and propionate supplementation. We also utilized *ex vivo* organotypic vagal cultures to investigate the signaling pathways downstream of propionate FFAR3 activation.

**Results:**

Vagal-FFAR3KO led to increased meal size in males and females, and increased food intake during fasting/refeeding and WD challenges. In addition, the anorectic effect of propionate supplementation was lost in vagal-FFAR3KO mice. Sequencing approaches combining *ex vivo* and *in vivo* experiments revealed that the cross-talk of FFAR3 signaling with cholecystokinin (CCK) and leptin receptor pathways leads to alterations in food intake.

**Conclusion:**

Altogether, our data demonstrate that FFAR3 expressed in vagal neurons regulates feeding behavior and mediates propionate-induced decrease in food intake.

## Highlights

• Lack of vagal FFAR3 increases food intake.
• Anorectic effect of propionate is lost when FFAR3 is absent from vagal neurons.
• FFAR3 signaling cross-talks with cholecystokinin (CCK) and leptin receptor pathways to alter food intake.

## 1 Introduction

Rates of obesity and the associated metabolic diseases/disorders continue to rise, and new treatment strategies and dietary interventions are needed to combat this epidemic [1,2]. The “Western diet” (WD), consisting of highly processed foods rich in saturated fat, sugar, and cholesterol, while low in fiber, likely contributes to the obesity epidemic [3]. Recent work has revealed the negative effects that the WD can have on the gut microbiota, and emphasis has been placed on understanding how gut-microbiota disruption modifies neural control of energy balance and glucose homeostasis [4,5]. Gut-microbiota fermentation of dietary fiber produces short-chain fatty acids (SCFAs), which have been proposed to improve host metabolic health through various mechanisms [6,7], including lowering food intake *via* the vagus nerve [8,9]. For instance, *Lactobacillus* strains have been commercially developed as probiotics that can increase SCFA production [10], and both *Lactobacillus* and SCFAs may exert effects on the central nervous system (CNS) *via* the vagus nerve [7,11,12].

The vagus nerve (cranial X) connects the brain and the visceral organs, providing a bidirectional line of communication to control homeostatic functions, including feeding [13]. The majority of vagal neurons are sensory (afferents), with cell bodies located in the nodose ganglia (NG). Vagal sensing of meal size, caloric content, gastrointestinal (GI) hormones, and nutrient composition fine-tunes an appropriate physiological and behavioral response to a meal [[14], [15], [16]]. Surgical disruption of the vagus nerve disrupts the feeding behavior [17] and eliminates the anorectic effect of fibers and SCFAs [8]; however, the molecular mechanisms of this signaling have yet to be revealed [8,9]. The SCFAs acetate, propionate, butyrate, and valerate can signal through multiple g-protein-coupled receptors (GPCRs) including hydroxycarboxylic acid receptor 2 (HCAR2), olfactory receptor 78 (Olfr78), and free fatty acid receptors 2 (FFAR2) and 3 (FFAR3) [18]. We investigated the expression patterns of these GPCRs in vagal neurons to determine how SCFAs regulate feeding behavior. In the current study, we show *Ffar3* expression in NG neurons innervating throughout the gut. We developed a mouse model for cell-type-specific deletion of *Ffar3* and sought to investigate whether FFAR3 expressed in vagal neurons mediates gut microbiome–brain regulation of feeding behavior in lean and obese mice. We show for the first time that vagal neuron expression of *Ffar3* is necessary for normal feeding behavior, and genetic deletion of this SCFA-binding receptor eliminates the positive benefits of propionate supplementation on energy balance.

## 2 Methods and materials

### 2.1 Animal studies

Animal studies were conducted in accordance with the recommendations of the Guide for the Care and Use of Laboratory Animals of the National Institutes of Health (cite) and with the approval of the Loyola University Chicago Institutional Animal Care and Use Committee. C57BL/6J (#000664), RiboTag (#011029), Phox2b-cre (#016223), and Vglut2-ires-cre (#028863) were obtained from Jackson laboratory (Maine, USA). Mice were group housed and kept in a 12:12 h light/dark cycle at 22–25 °C. Mice received either NC (Teklad LM-485) or WD (TD88137, Teklad Diets; 42% kcal from fat, 34% sucrose by weight, and 0.2% cholesterol total) (Envigo, Indiana, USA) starting at 7 weeks of age. Body weights (BW) were recorded weekly from weaning.

### 2.2 Generation of FFAR3 flox mice

FFAR3 flox mice were generated using the Targeted Knockout First, Reporter-tagged Insertion with Conditional Potential (conditional-ready) strategy by the International Knockout Mouse Consortium. The critical portion of exon 2 was flanked by loxP sites, with upstream elements including FRT – LacZ – loxP – neo – FRT. Flp-mediated recombination converted the mutated allele to the conditional allele. *In vitro* fertilization (IVF) was performed at Jackson Laboratories to generate FFAR3 flox mice on the C57BL/6J background, and subsequent breeding was carried out at Loyola University, Chicago.

### 2.3 Indirect calorimetry and meal pattern analysis

Mice were individually housed for indirect calorimetry and feeding behavior assessment using the TSE Phenomaster home-cage system (TSE systems, Chesterfield, MO). Ambient temperature was maintained at 24.5 °C for all studies using a Phenomaster climate chamber. All experimental groups were BW matched upon the start of metabolic assessment to avoid confounding of BW on energy balance [19], as described previously [20,21]. Furthermore, ANCOVA with BW as the covariate was used to analyze energy expenditure, as suggested by Tschöp et al. [19]. All food and water intake data shown were collected using Phenomaster cages. A meal was defined as a bout of feeding larger than 0.05 g with a 15-minute intermeal interval when no further food removal occurred [22]. Meal sizes were then broken down into categories: small (<0.09), medium (>0.09–0.13), large (>0.13–0.18), very large (>0.18) as described by Zhang et al. [23].

### 2.4 Fasting/refeeding and CCK injection

After overnight fast (18:00–8:00) in Phenomaster cages with free access to water, food was replenished, and mice were fed *ad libitum*. For CCK studies, after overnight fast, mice received intraperitoneal injection (i.p.) of 1 μg/kg body mass CCK8s (Tocris) or saline 20 min before food was replaced in feeders.

### 2.5 Propionate supplementation

25 mg/mL sodium propionate (Millipore Sigma) or equimolar saline was administered in drinking water *ad libitum*. Water was changed every other day. The daily water intake was multiplied by the concentration of propionate in the water and then divided by body mass to approximate the dose at 1.8 g propionate/kg body mass. The lean group received propionate supplemented water at the same time diet was switched to WD (in metabolic cages), and the DIO group continued with WD feeding when water was switched.

### 2.6 Glucose tolerance test (GTT) and fasting insulin

Overnight (14 h) fasted mice were given i.p. dose of glucose (1 g/kg BW) after measuring their fasting glucose levels. Their blood glucose levels were then monitored using AlphaTrak glucometer for rodents (Fisher Scientific, Pennsylvania, USA). Fasting blood was collected in and centrifuged at 2,000×*g* for 10 min, and Insulin ELISA (Sigma–Aldrich EZRMI-13K) was performed according to the manufacturer's instructions.

### 2.7 Cecal DNA isolation and 16S sequencing

The cecal contents were collected, and an equal amount of cecal content was homogenized and DNA isolated using the QIAamp Powerfecal DNA Kit (Qiagen). qPCR was performed with universal 16S primers. The Loyola Genomic Core was used for performing PCR of 16S rRNA V4-5 regions sequenced by the Illumina HiSeq4500 platform, as done previously (cite); 16S sequences were aligned using the Silva Taxonomy Annotation, and the files were uploaded to MicrobiomeAnalyst [24,25] for analysis (https://www.microbiomeanalyst.ca/).

### 2.8 Plasma SCFA quantification

After decapitation under anesthesia, blood was collected in K3EDTA tubes (Sarstedt) and centrifuged at 2,000×*g* for 10 min. The LC/MS analysis was performed on AB Sciex Qtrap 5500 coupled to the Agilent UPLC/HPLC system. All of the samples were analyzed in triplicate by the University of Illinois at Chicago Mass Spectrometry Core and an internal control was used to evaluate the interassay variability.

### 2.9 Hypothalamic and intestinal RNA isolation and qPCR

Whole hypothalamus, duodenum, and colon tissue was homogenized using Trizol (Invitrogen) in Bullet Blender bead tubes (Next Advance). After BCP (Sigma) phase separation, RNA was purified in Zymo columns with DNAse I treatment according to the manufacturer's instructions. RNA was converted to cDNA with the High-Capacity Reverse Transcription Kit (Life Technologies), and qPCR was performed in triplicate with gene expression normalized to 18s.

### 2.10 Single cell target sequencing data utilization

RNA-sequencing data from Bai et al. [14] target-scSeq was downloaded from Gene Expression Omnibus ID GEO: GSE138651. Ln(cpm) > 1 was considered “positive” for a given gene.

### 2.11 Chromogenic *in situ* hybridization

Chromogenic *in situ* hybridization for *Ffar3* (ACD Probe cat#447011) was done exactly as described previously [26] using a combination of chromogenic RNAScope (FastRed) and GFP immunohistochemistry.

### 2.12 Immunohistochemistry

NG sections (18 μM) from RiboTag+/+:Nav1.8Cre+/-, RiboTag+/+:Vglut2-Cre+/-, and control mice were probed for HA-tag (Biolegend, #901513, California, USA; secondary-goat anti-mouse 647, ab150115, Abcam, Massachusetts, USA) as described before [27,28].

### 2.13 Vagal explants

After weaning, the mice were euthanized *via* cervical dislocation under isoflurane anesthesia and decapitated. Left and right nodose ganglia were exposed, and the vagus nerve was cut to remove the intact ganglia. Ganglia were cultured on open air-interface membrane inserts (Millicell). After three days in culture, NG were serum starved (MEM, 2.5 mM GlutaMax, 2.5% horse serum) for 12 h before propionate treatment (1 mM in low serum media). NG were then stored at −80 °C before RNA isolation.

### 2.14 RNA isolation, cDNA library construction, and illumina sequencing

Total RNA was extracted from the NG of mice using Arcturus PicoPure RNA Isolation Columns (Thermo Fisher). The Northwestern Genomic Core (NuSeq) was used for performing the experiments and biostatistical analyses. Briefly, full-length cDNA synthesis and amplification were carried out with the Clontech SMART-Seq v4 Ultra Low Input RNA Kit. Subsequently, Illumina sequencing libraries were prepared from the amplified full-length cDNA with the Nextera XT DNA Library Preparation Kit. The sequencing of the libraries was conducted on an Illumina NextSEq 500 NGS System. Single 75-bp reads were generated with dual indexing. RNA sequencing analysis was done with STAR and DESeq2. The quality of reads, in FASTQ format, was evaluated using FastQC. Pathway analysis was performed using the PANTHER classification system (www.pantherdb.org). Putative transcription factors were predicted using the oPOSSUM single site analysis tool (http://opossum.cisreg.ca).

### 2.15 Vagal primary cultures

Left and right NG from FFAR3 flox and vagal-FFAR3KO mice (5 weeks or younger) were dissociated in trypsin collagenase using glass pipettes. NG neurons were plated on poly-l-lysine-coated dish in DMEM/F12 with AraC and pen/strep. After 3 days in culture, the primary neurons were serum starved overnight in empty DMEM/F12, and the media was collected and frozen at −80 °C. CART levels were measured using the CART (61–102) EIA Kit (Phoenix Pharmaceuticals).

### 2.16 Statistics

Statistical analyses were performed using GraphPad Prism 9. Student's t-test, multiple t-tests, and two-way ANOVA were performed to assess the differences between the groups, and time or genotype served as co-variants. Fischer's LSD was used for all *post-hoc* analyses. The statistical test and sample size are indicated in figure legends for all experiments. All data are expressed as means, and the error bars indicate SEM. For all the experiments, males and females were analyzed separately. Randomization and blinding were not used. Energy expenditure was analyzed using ANCOVA with body mass as the co-variate using Origin Pro. The statistical significance was indicated by ∗*p* < 0.05, ∗∗*p* < 0.01, and ∗∗*p* < 0.001.

## 3 Results

### 3.1 Vagal FFAR3 knockout disrupts meal-induced satiation

**Figure 1:**
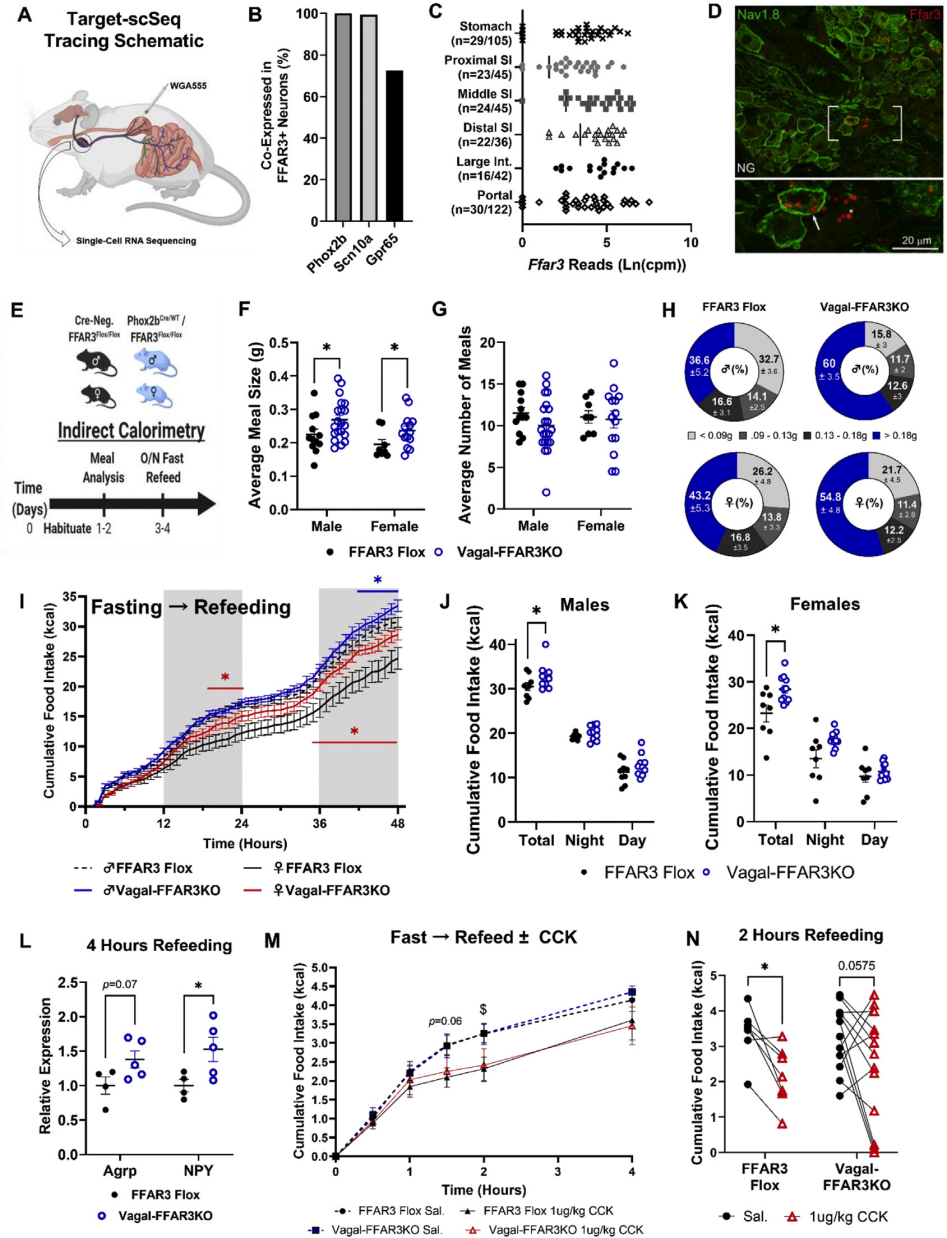
Vagal FFAR3 knockout disrupts meal-induced satiation. (A–C) Characterization of the *Ffar3* innervation pattern and co-expression from target single-cell (Target-scSeq.) RNA sequencing (Bai et al., 2019). Tracing schematic showing retrograde membrane dye labeling vagal afferents from different intestinal regions (A). Percent co-expression of other nodose ganglia (NG) markers within *Ffar3*+ neurons (B). *Ffar3* expression across vagal neurons innervating different parts of the intestine (C). (D) Chromogenic *in situ* hybridization staining *Ffar3* mRNA (red) in Nav1.8-cre-ChR2 positive (green and arrow) and Nav1.8-negative (no stain and asterisk) vagal sensory neurons. (E) Experimental paradigm for panels F–L. A similar paradigm was used for panels M−N but with CCK8s or saline injection after overnight fasting and prior to refeeding. (F–H) Average nightly meal size (F), number of meals (G), and relative percentage of different meal sizes (H) (*n* = 12–20 males/group and 8–14 females/group, two-way ANOVA, Fischer's LSD ∗*p* < 0.05). (I–K) Cumulative food intake after overnight fast in male and female FFAR3 flox and vagal-FFAR3KO mice (multiple paired *t*-tests, blue asterisk compares males FFAR3 flox vs Vagal-FFARKO, red asterisk compares females, *n* = 8–10 males/group and *n* = 8–12 females/group). (L) Whole hypothalamic gene expression of the orexigenic neuropeptides *Agrp* and *Npy* (*n* = 4–5 females/group, Student's *t*-test, ∗*p* < 0.05, gene expression relative to 18s and confirmed with β-actin). (M−N) Cumulative food intake trace of lean NC-fed mice after i.p. saline or 1 ug/kg CCK8s (M) (multiple paired *t*-tests $*p* < 0.05 FFAR3 flox saline vs. FFAR3 flox CCK). Cumulative food intake two hours post-injection (N) (two-way ANOVA with repeated measures ∗*p* < 0.05). (*n* = 3 female, 4 male FFAR3 flox mice; 4 female, 9 male vagal-FFAR3KO mice). Error bars indicate mean ± SEM.

First, we used a ribosome-tagging strategy to determine whether SCFA-binding GPCRs were translated in NG sensory neurons. Our data revealed that *Ffar3* was expressed and actively translated, along with olfactory receptor 78 (*Olfr78*) to a lesser degree (Supp. Figure 1A). To better understand the innervation and gene expression patterns of *Ffar3*+ neurons, we analyzed single-cell target RNA sequencing (Target-scSeq.) data sets published by Bai et al. [14], as represented in Figure 1A. According to our secondary analyses, 37% of all *Phox2b*+ NG neurons (marker of all vagal neurons) expressed *Ffar3* (Supp. Figure 1B). All *Ffar3*+ neurons co-expressed *Phox2b*, 99% co-expressed *Scn10a* (marker of small-diameter peripheral afferents), and 70% co-expressed *Gpr65* (marker of mucosal innervation patterns) (Figure 1B). *Ffar3* mRNA was detected in NG neurons tracing back from the stomach, small and large intestine, and portal vein (Figure 1C). Furthermore, *Ffar3* expression was detected in NG neuronal populations known to control feeding, including 56% of oxytocin receptor (*Oxtr*), 45% of glucagon-like peptide receptor 1 (*Glp1r*), 40% of leptin receptor (*LepR*), and 28% of *Cckar*+ neurons (Supp. Figure 1B) [14,16]. Consistent with the Target-scSeq. data, we identified *Ffar3* in both Nav1.8 (*Scn10a*)-positive and -negative neurons *via* chromogenic *in situ* hybridization (CISH) (Figure 1D). *Ffar3* mRNA, however, was not detected in the dorsal vagal complex (DVC) *via* CISH (Supp. Figure 1C), in agreement with previous studies showing that *Ffar3* is not present in the CNS [29].

Many conflicting studies have reported the roles of SCFAs and FFAR3 in the control of glucose homeostasis and feeding behavior [[7], [8], [9],^30^]. Cell-specific roles are likely masked in whole body KO studies, and cell-specific studies are absent from the literature; therefore, we generated mice with loxP sites flanking the exon 2 region of *Ffar3* (Supp. Figure 1D). We crossed phox2b-cre heterozygous mice (*phox2b*^Cre/WT^) with *Ffar3* flox homozygous (*Ffar3*^Fl/Fl^) mice to generate vagal FFAR3 knockout mice (Vagal-FFAR3KO) and FFAR3 flox littermate controls (Figure 1E). As expected, we did not detect *Ffar3* transcripts in the NG of *phox2b*^Cre/WT^/*Ffar3*^Fl/Fl^ (Supp. Figure 1E and H), but *Ffar3* expression remained intact in the NG of *Ffar3*^Fl/Fl^ controls (Supp. Figure 1E and G). Importantly, our *phox2b*^Cre/WT^/*Ffar3*^Fl/Fl^ model also did not diminish intestinal *Ffar3* expression (Supp. Figure 1F). To determine whether the loss of FFAR3 in vagal neurons disrupted the feeding behavior or energy balance, we performed indirect calorimetry on male and female mice at 8–9 weeks old (Figure 1E). When fed NC *ad libitum*, male and female vagal-FFAR3KO mice consumed larger meals compared with their FFAR3 flox littermates (Figure 1F), with a larger percentage of “very large meals” (over 0.18 g) observed in males (Figure 1H). Many male vagal-FFAR3KO mice compensated for the increased meal size by reducing the number of meals (Figure 1G), resulting in equal cumulative food intake compared with FFAR3 flox controls (Supp. Figure 1I). On the other hand, female mice lacking vagal FFAR3 consumed an equal number of meals as the control littermates, resulting in an increased cumulative food intake at baseline (Supp. Figure 1J).

Given the apparent deficit in meal-induced satiation caused by the genetic ablation of vagal *Ffar3*, we challenged FFAR3 flox and vagal-FFAR3KO mice with overnight fasting and quantified refeeding over 48 h. Female vagal-FFAR3KO mice consumed more calories within the first 24 h (Figure 1I), while male vagal-FFAR3KO mice showed increased food intake during the second day of refeeding (Figure 1I). In both sexes, vagal-FFAR3KO mice consumed more calories over 48 h after an overnight fast (Figure 1I–K). Loss of vagal FFAR3 also caused a trend toward increased water consumption (Supp. Figure 1L). Consistent with the idea that vagal FFAR3 relays meal termination signals to the hypothalamus [8], hypothalamic gene expression of the orexigenic neuropeptides agouti-related peptide (*Agrp)* and neuropeptide Y (*Npy)* were elevated 4 h after the reintroduction of food in vagal-FFAR3KO mice compared with that observed in FFAR3 flox controls (Figure 1L). Hypothalamic *Npy* and *Agrp* mRNA are upregulated during fasting and subsequently downregulated during feeding [31]. Thus, our data suggest that SCFA sensing *via* vagal FFAR3 may contribute to post-absorptive downregulation of these orexigenic transcripts to terminate feeding.

Interestingly, male mice lacking vagal FFAR3 compensated for larger food intake by elevating energy expenditure (EE) (Supp. Figure 1M and O), without changing ambulatory activity (Supp. Figure 1K), resulting in a net energy balance equal to FFAR3 flox controls (Supp. Figure 1P) and no subsequent difference in body mass (Supp. Figure 1Q). In contrast, female vagal-FFAR3KO mice did not exhibit increased EE (Supp. Figure 1N–O), resulting in a higher energy balance during refeeding (Supp. Figure 1P), potentially indicating sex differences in diet-induced thermogenesis. We did not detect any differences in glucose tolerance, fasting glucose, or insulin levels in lean female vagal-FFAR3KO mice or DIO male vagal-FFAR3KO mice (Supp. Figure 1R–U).

Given the established role of vagal CCK signaling in the proper regulation of feeding and because vagal neurons exhibit cooperative signaling of GI hormones [[32], [33], [34]], we wondered whether FFAR3 deletion might alter CCK sensitivity. To test this, we injected (i.p.) male and female mice with saline or low dose (1 μg/kg) CCK-8s after overnight fasting. We then measured the subsequent refeeding behavior. CCK-8s significantly lowered food intake in FFAR3 flox controls, whereas vagal-FFAR3KO mice appeared less responsive (Figure 1M−N), suggesting that vagal FFAR3 signaling contributes to CCK sensitivity.

### 3.2 Western diet disrupts microbiome composition, lowering circulating propionate

**Figure 2:**
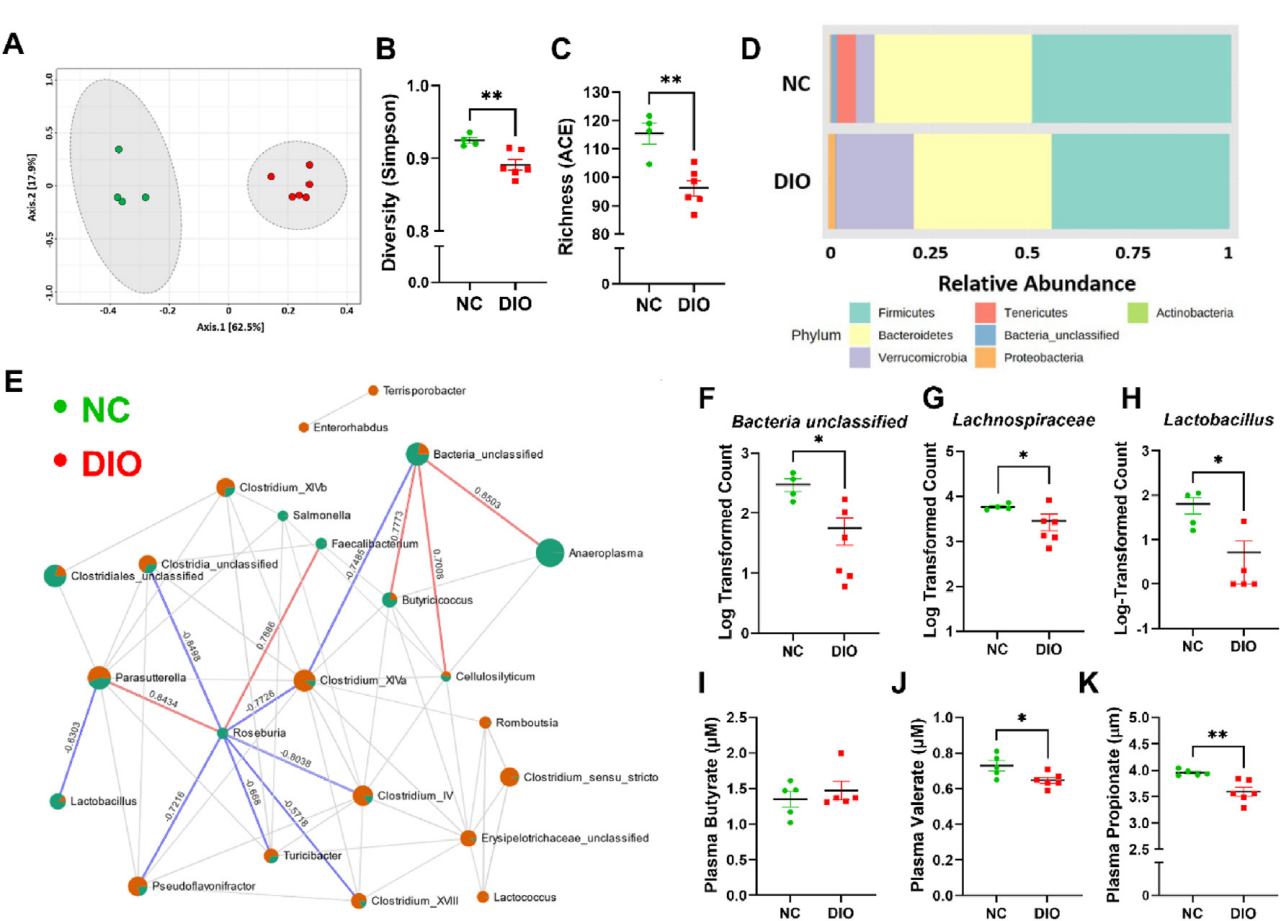
DIO disrupts the microbiome composition, lowering circulating propionate. (A–H) 16S sequencing of DNA isolated from the cecal contents of DIO and NC littermates. NC vs. DIO principal component analysis (PCA) (A), Simpson diversity (B) and ACE richness indices (C), relative phyla abundances (D), and SparCC correlation network analysis (E) (*n* = 4–6 males/group, zero-inflated Gaussian Fit FDR-adjusted ∗*p* < 0.05). Log-transformed actual counts of Unclassified (F), Lachnospiraceae (G), and *Lactobacillus* (H) genera (*n* = 4–6 males/group, SparCC correlation analysis line indicates *R* > 0.5, *p* < 0.05). (I–K) Circulating SCFA concentrations (μM) of butyrate (I), valerate (J), and propionate (K) (*n* = 5–6 mice/group, Student's *t*-test, ∗*p* < 0.05, ∗∗*p* < 0.01).

Since FFAR3 binds SCFAs [18] produced by gut microbes, we analyzed the microbiome composition and circulating SCFA levels of diet-induced obese (DIO) mice. We fed 7-week-old male mice with WD for 12 weeks and performed 16S sequencing on DNA isolated from cecal contents. We found that DIO mice exhibited altered gut microbiome composition compared with age-matched normal chow (NC)-fed littermates (Figure 2A, Supp. Figure 2A). DIO mice also exhibited reduced microbial diversity (Figure 2B, Supp. Figure 2B) and richness (Figure 2C, Supp. Figure 2C). The DIO gut microbiome was characterized by a reduction in Tenericutes and Unclassified Bacteria and an expansion of Proteobacteria and Verrucomicrobia Phyla (Figure 2D, Supp. Figure 2A). Sparse correlations for compositional data (SparCC) [35] network analysis demonstrated that *Roseburia* and *Butyricicoccus* were among the key determinants of the NC-fed microbial landscape (Figure 2E), as their abundance was negatively correlated with several other genera that were expanded in DIO mice. We also found that bacteria Unclassified (Figure 2F), Lachnospiraceae (Figure 2G), and *Lactobacillus* (Figure 2H) were among the top genera decreased in DIO mice (Supp. Figure 2A). Given the proposed role of *Lachnospiraceae*, *Lactobacillus*, *Roseburia*, and *Butyricicoccus* in producing SCFAs [36], we measured the plasma levels between NC-fed and WD-fed DIO mice. DIO did not alter butyrate or acetate levels (Figure 2I, Supp. Figure 2D) while significantly lowering circulating valerate (Figure 2J) and propionate (Figure 2K), which are known high-affinity ligands for FFAR3 [18].

### 3.3 Vagal-FFAR3KO increases WD-induced food intake and weight gain and eliminates the anorectic effect of propionate supplementation in DIO mice

**Figure 3:**
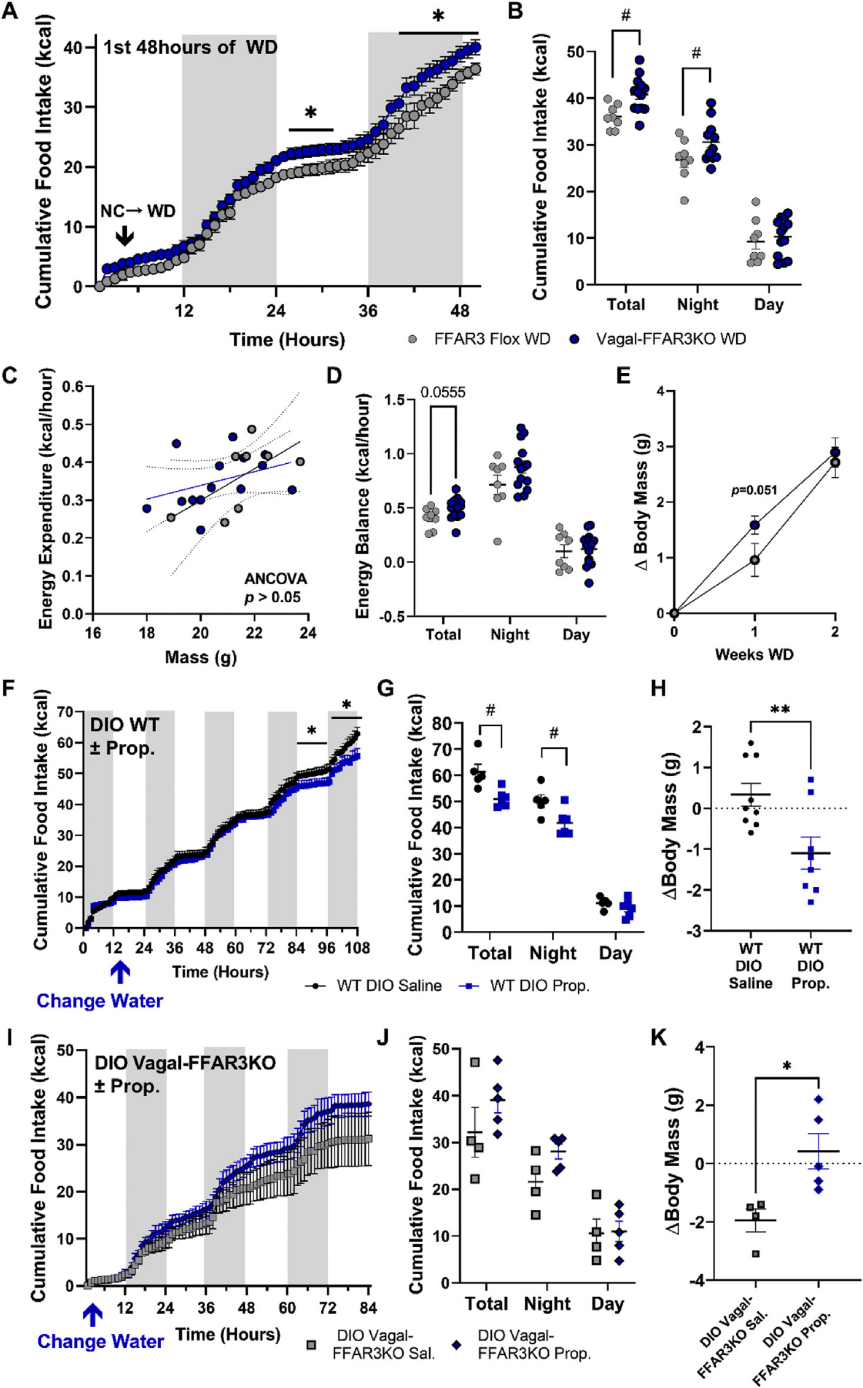
Vagal-FFAR3KO increases WD-induced food intake and weight gain and eliminates the anorectic effect of propionate supplementation in DIO mice. (A–D) Indirect calorimetry of lean FFAR3 flox and vagal-FFAR3KO mice during the first 48 hours of WD challenge. Hourly (A) and cumulative food intake (B), energy expenditure vs. body mass (C), and average energy balance (D) (multiple t-tests, ∗*p* < 0.05; two-way ANOVA with repeated measures, Fischer's LSD Multiple Comparison's #*p* < 0.05; *n* = 8–13 males/group). (E) Change in body mass after two weeks of WD feeding (Student's *t*-test, *n* = 8–17 male mice/group). (F–G) Hourly (F) and cumulative food intake (G) of WT DIO mice (fed WD at 9 weeks) after supplementation of saline or 25 mg/mL sodium propionate in drinking water (multiple t-tests, ∗*p* < 0.05; two-way ANOVA with repeated measures, Fischer's LSD Multiple Comparison's, *#p* < 0.05, *n* = 5–6 males/group). (H) Subsequent change in body mass of WT DIO mice after 1 week of propionate supplementation in drinking water (Student's *t*-test, ∗∗*p* < 0.01*, n* = 8–9 males/group). (I–K) Hourly (I) and cumulative food intake (J) of DIO Vagal-FFAR3KO mice (fed WD at 9 weeks) after supplementation of saline or 25 mg/mL sodium propionate in drinking water and the subsequent change in body mass (K) (*n* = 4–5 males/group). Error bars indicate mean ± SEM.

Since we found that genetic ablation of vagal *Ffar3* disrupted meal satiation, we wondered how vagal-FFAR3KO mice might respond to the challenge of a more energy-dense WD chow. To test this, we challenged lean male FFAR3 flox and vagal-FFAR3KO mice with WD while performing indirect calorimetry. Upon switching to WD, vagal-FFAR3KO mice consumed more food (Figure 3A–B) without significantly increasing energy expenditure (Figure 3C), resulting in a trends toward larger positive energy balance (Figure 3D) and a transient increase in weight gain (Figure 3E).

Previous studies have demonstrated the positive effects of propionate and other SCFAs in reducing food intake [8,37]; therefore, we investigated the contribution of vagal FFAR3 to SCFA-induced decrease in food intake in our WD-induced obesity model. As a proof-of-principle study, we supplemented 25 mg/mL sodium propionate in the drinking water of 7-week-old lean WT mice at the same time that their food was switched from NC to WD. We chose drinking water as the route of administration to avoid the possible confounding of altered food palatability. Propionate led to reduced water intake to approximately 2 mL per day (Supp. Figure 3A), with an estimated dose of 1.8 g propionate per kg body mass. This dose was sufficient to lower food intake in lean male mice upon switching from NC to WD (Supp. Figure 3B), preventing WD-induced weight gain (Supp. Figure 3C) with nearly zero fat mass gain after one-week WD-feeding (Supp. Figure 3D). Next, we fed a separate cohort of WT mice with WD for 9 weeks to induce diet-induced obesity and then supplemented the drinking water with sodium propionate (25 mg/mL) or equimolar saline. Again, propionate led to reduced food intake (Figure 3F–G) and water intake (Supp. Figure 3E) without altering the energy expenditure (Supp. Figure 3F), inducing a loss in body mass (Figure 3H, Supp. Figure 3G) and fat mass (Supp. Figure 3H).

Having established the anorectic effect of propionate in lean and DIO WT mice, we next examined how vagal-FFAR3KO mice responded to the same paradigm of propionate supplementation. After 9 weeks of WD-feeding, male DIO mice lacking vagal FFAR3 were supplemented with either 25 mg/mL sodium propionate or equimolar saline in the drinking water. Strikingly, propionate supplementation failed to lower food intake (Figure 3I–J) and water intake (Supp. Figure 3E) compared with saline controls. Without altering EE (Supp. Figure 3J), propionate supplementation caused a more positive energy balance (Supp. Figure 3J) and weight gain (Figure 3K, Supp. Figure 3L). Altogether, we found that genetic ablation of vagal *Ffar3* disrupted ingestive behavior in a variety of contexts, and the anorectic effect of propionate was dependent upon the expression of FFAR3 in vagal neurons. We next explored the signaling pathways through which propionate and FFAR3 activation might be acting in vagal neurons.

### 3.4 Propionate signals through FFAR3-dependent and independent pathways in the nodose ganglion

**Figure 4:**
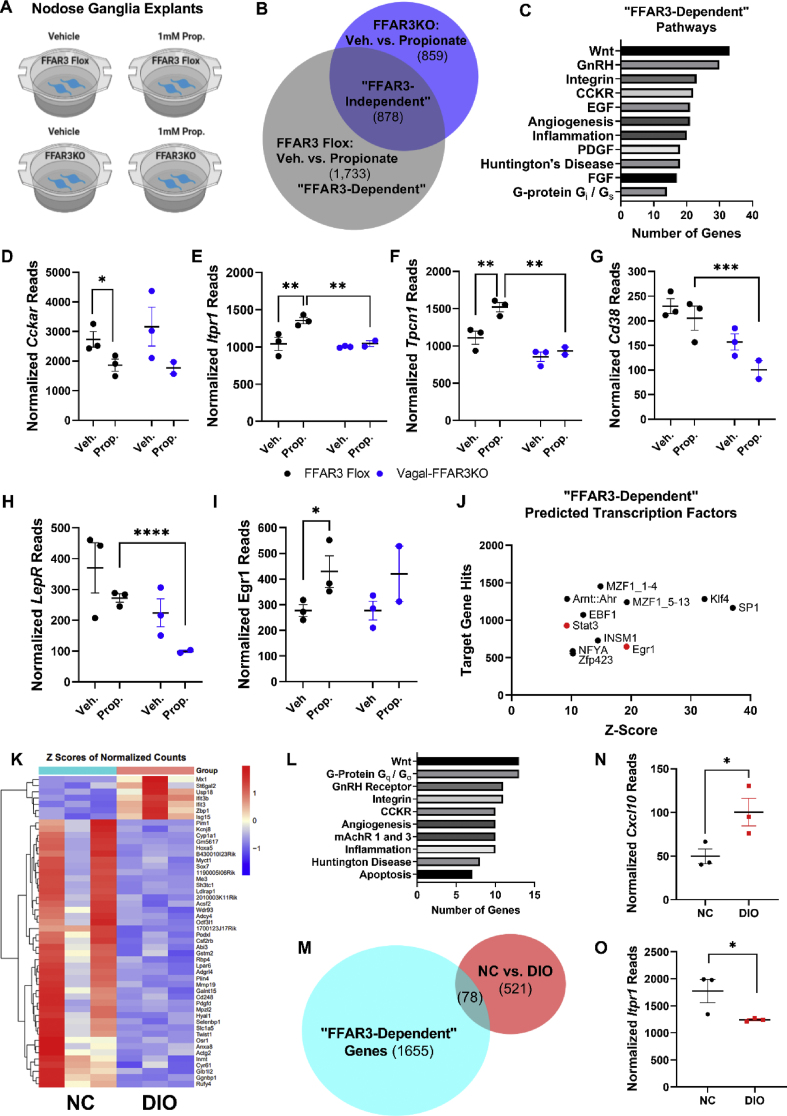
Propionate signals through FFAR3-dependent and independent pathways in the nodose ganglion. (A) Graphical depiction of the experimental paradigm. Nodose ganglion explants were cultured from FFAR3 flox or vagal-FFAR3KO mice and stimulated with vehicle or 1 mM propionate for 12 hours; RNA was isolated and low-input sequencing was performed. (B) Venn diagram demonstrating the number of genes with expression significantly different between the treatment groups. (C) PANTHER pathway classification of genes that were altered in FFAR3 flox NG after propionate treatment but not vagal-FFAR3KO NG treated with propionate. (D–I) RNA sequencing results of genes within the “CCKR signaling pathway” (P06959) (D–G), leptin receptor (H), and early growth response 1 (I). (J) Prediction of altered transcription factors of “FFAR3-indendent” group (see Figure 3B) using the oPOSSUM database (Ho Sui et al., 2005). (K–L) NC vs. DIO top 50 genes (K) and altered PANTHER pathways (L). (M) Venn diagram showing overlapping “FFAR3-dependent” (see Figure 3B) and NC vs. DIO groups. (N–O) RNA sequencing results for C-X-C motif chemokine ligand 10 (N) and Inositol 1,4,5-Trisphosphate Receptor Type 1 (O). Error bars indicate mean ± SEM, *n* = 2–3 nodose ganglia pairs/treatment replicate, FDR-adjusted ∗*p* < 0.05.

To investigate the signaling pathways induced by propionate and downstream of FFAR3 within the vagal neurons, we cultured NG explants from FFAR3 flox and vagal-FFAR3KO mice and treated them with either vehicle or sodium propionate (Figure 4A). Propionate directly altered a total of 2611 transcripts in FFAR3-expressing (FFAR3 Flox) NG, and 1737 transcripts were differentially expressed in vagal-FFAR3KO NG after propionate treatment (Figure 4B, Supp. Figure 4A and B). To gain an insight into FFAR3-dependent pathways, we analyzed the list of transcripts altered by propionate in FFAR3-expressing ganglia, which were unchanged by propionate in vagal-FFAR3KO mice (Figure 4B). PANTHER (Protein Analysis through evolutionary relationships) Classification System [38] of these 1733 “FFAR3-dependent” transcripts revealed Wnt, GnRH, and integrin to be the top three signaling pathways downstream of propionate-FFAR3 activation (Figure 4C), and these were also among the top “FFAR3-independent” pathways altered by propionate (Supp. Figure 4C). Consistent with our data suggesting reduced CCK sensitivity in vagal-FFAR3KO mice (Figure 1M and N), several transcripts within the “CCKR” signaling pathway were altered by propionate (Figure 4C–G), including *Cckar, Itpr1, Tpcn1,* and *Cd38*. In addition, *Lepr* transcripts were reduced in propionate-treated vagal-FFAR3KO NG (Figure 4H, Supp. Figure 4B). We utilized the oPOSSUM database for the prediction of transcription factors (TFs) from our RNA sequencing results [39], and many of the “FFAR3-dependent” transcripts had signal transducer and activator of transcription 3 (STAT3) and early growth response 1 (Egr1) as the predicted TFs (Figure 4J). *Egr1* expression was increased in FFAR3-expressing NG after 12-hour propionate treatment (Figure 4I) and after 2-h stimulation in sensory neurons isolated from RiboTag mice (Supp. Figure 4D and E). Previous studies have demonstrated that leptin increases *Egr1* expression, and CCK induces translocation to the nucleus, ultimately potentiating the upregulation of the transcript for the satiety peptide cocaine–amphetamine-regulated transcript (CART) [32]. Consistent with this possibility, we found that primary neurons isolated from vagal-FFAR3KO NG exhibited decreased secretion of CART, compared with FFAR3 flox primary neurons (Supp. Figure 4F). The potential cross-talk between FFAR3, leptin, and/or CCK receptors in vagal neurons is intriguing and requires further study.

Finally, we performed RNA sequencing on NG isolated from NC vs. DIO mice (Figure 4K–O). Compared with NC-fed controls, DIO significantly altered 599 genes (top 50 shown in Figure 4K), again many falling in Wnt, GnRH, Integrin and CCKR signaling pathways (Figure 4L). Given that DIO reduced circulating propionate (Figure 1K), we investigated whether any transcripts altered in DIO mice were also “FFAR3-dependently” altered by propionate. We identified 78 overlapping transcripts between “NC vs. DIO” and “FFAR3-dependent” groups (Figure 4M). Interestingly, several transcripts within the “inflammation mediated by chemokine and cytokine signaling pathway” (P00031) were upregulated in DIO mice and downregulated *via* propionate, including *Cxcl10, Irf7, Ifi44,* and *Ifi3* (Figure 4M and N, Supp. Figure 4G). Propionate also appeared to alter the expression of inflammation signaling pathway genes in an “FFAR3-independent” (Supp. Figure 4C) manner. Again, many transcripts altered in DIO mice had Stat3 and Egr1 as the predicted transcription factors (Supp. Figure 4H) and fell into the CCKR signaling map pathway (Figure 4L). Expression of Serca3 (*Atp2a3*) and the IP3-receptor (*Itpr1*) were decreased in DIO NG (Figure 4O, Supp. Figure 4G), potentially indicating altered upstream CCKaR or other Gq-mediated signaling. The heterogeneity of localization and co-expression patterns of *Ffar3*+ vagal neurons (Supp. Figure 2B) open the possibility of cooperative signaling with several key GI hormones that control feeding, such as GLP1, CCK, and leptin.

## 4 Discussion

We utilized several methods to characterize the expression patterns of SCFA-binding GPCRs in nodose ganglia. We performed RNA sequencing on whole nodose ganglia, ribotag qPCR of vagal sensory neurons, and analysis of single-cell data sets of vagal sensory neurons. In the RNA sequencing data of whole nodose ganglia, which also include transcripts from non-vagal cells, we detected the expression of all four receptors *Ffar3*, *Olfr78*, *Ffar2*, and *Hcar2*. However, in our ribotag data, we were only able to detect *Ffar3* and *Olfr78* being actively translated in vagal sensory neurons. In support of this, the secondary analysis of single-cell nodose neuron data also showed that ∼37% of vagal neurons express *Ffar3* and ∼30% express *Olfr78*; only one neuron was detected expressing *Ffar2*, and one neuron expressed *Hcar2*. Two separate single-cell analyses identified Olfr78 as a marker of a unique population of vagal sensory neurons [14,40]. Olfr78 also binds lactate, and this signaling contributes to the proper regulation of respiration [41]. Thus, future studies assessing the specific role of *Olfr78*+ vagal neurons innervating the lungs and heart would be of particular interest. Although we detected low and variable expression of *Ffar2* and *Hcar2*, future studies may reveal important roles for these receptors in vagal neurons and/or non-neuronal cells residing in nodose ganglia.

Whole-body knockout studies have generated conflicting data regarding the role of FFAR3 in controlling the energy balance. For instance, Samuel et al. found that global FFAR3KO decreased energy harvest (reduced caloric and SCFA absorption) and decreased adiposity [42]. In contrast, multiple studies have reported the role of global knockout in reducing energy expenditure and increasing adiposity [43,44]. Thus, it is likely that FFAR3 signaling regulates multiple components of energy balance through different cell types, which emphasizes the urgent need for cell-type specific studies. We discovered that FFAR3 in vagal sensory neurons regulates short-term feeding and drinking behavior. WD challenge caused a transient increase in weight gain in vagal-FFAR3KO mice. Furthermore, we found that propionate supplementation in drinking water reduced food and water intake, dependent upon vagal FFAR3. Although disrupted vagal signaling is not thought to primarily cause diet-induced obesity, several studies have shown diminished vagal sensitivity to key satiety hormones (i.e. GLP1 and CCK) in obesity [45,46]. There is also substantial evidence that the vagus nerve can be therapeutically targeted to combat obesity [13,17,47]. Thus, although dysfunctional vagal FFAR3 signaling is not likely a causal factor in obesity, the vagal-FFAR3-dependent anorectic effect of propionate endows it with therapeutic potential.

Our RNA sequencing and food intake data indicated that the cross-talk between FFAR3 signaling and CCK and leptin receptors in vagal neurons leads to modifications in food intake. CCKaR signaling induces extracellular calcium influx in NG neurons [48], likely altering the gating of information to the CNS. Deletion of leptin receptors from vagal sensory neurons reduces satiation and CCK sensitivity, resulting in increased fat mass gain [49]. Furthermore, other studies have demonstrated the synergistic actions of CCK and leptin in vagal calcium signaling, peptide secretion, and long-term feeding behavior [[32], [33], [34],^49^]. Our data suggest that propionate/FFAR3 may also be involved in this synergistic interaction.

*Ffar3* expression was also found in a high percentage of*Glp1r*+ and *Oxtr*+ vagal neurons, which are populations known to control feeding and drinking behavior [14,50]. Activation of these GPCRs triggers neuronal excitation and neurotransmitter and/or peptide release through intracellular and extracellular calcium pathways that we found altered in our sequencing data [[51], [52], [53]]. Vagal-FFAR3KO mice drank more water during fasting/refeeding, and propionate reduced water intake in WT mice; however, it failed to do so in vagal-FFAR3KO mice. Given the overlap of the *Ffar3* expression in *Oxtr*+ neurons controlling water intake [14], it is likely that FFAR3 signaling contributes to the vagal control of thirst satiation.

Vagal sensory neurons synapse onto the dorsal vagal complex (DVC) where they release glutamate and other neuropeptides to control feeding [15]. Thus, future investigation would be needed to fully decipher how FFAR3 impacts calcium dynamics and peptide secretion into the brainstem to regulate satiety. Future studies are required for elucidating the precise signaling mechanisms of FFAR3 within the vagal sensory neurons, which might help develop effective therapies for the modulation of eating and drinking behavior. Ultimately, vagal satiation signals are integrated in the hypothalamus to control feeding behavior. Li et al. have demonstrated that butyrate decreases food intake by attenuating the activation of NPY+ neurons in the arcuate nucleus [8]. Our study supports the idea that vagal-FFAR3 signaling contributes to this process. Further work mapping the neurocircuitry of FFAR3+ vagal neurons may improve our understanding of how fiber fermentation *via* gut microbiota impacts post-absorptive satiety signals.

Recent elegant work has demonstrated links between maternal gut microbiota and nervous system development and behavior, and the vagus nerve has been suspected to mediate this connection *via* unknown mechanisms [54,55]. It is possible that we have uncovered a role for vagal FFAR3 in nervous system development that ultimately influences adult feeding behavior. Future work directly testing this hypothesis and utilizing conditional or chemogenetic models to temporally manipulate vagal FFAR3 may provide interesting insights into the role of FFAR3 in nervous system development and function. Our work stresses the need for further development of tissue-specific genetic tools to elucidate the precise mechanisms by which FFARs, fibers, and SCFAs control digestion, energy balance, glucose homeostasis, and behavior.
