# Supplementary material for: Vagal neuron expression of the microbiota-derived metabolite receptor, free fatty acid receptor (FFAR3), is necessary for normal feeding behavior
Source: Mol Metab. 2021 Oct 6;54:101350. doi: 10.1016/j.molmet.2021.101350 (PMC8567301; doi:10.1016/j.molmet.2021.101350)
Supplement: Multimedia component 1 [file mmc1.docx]

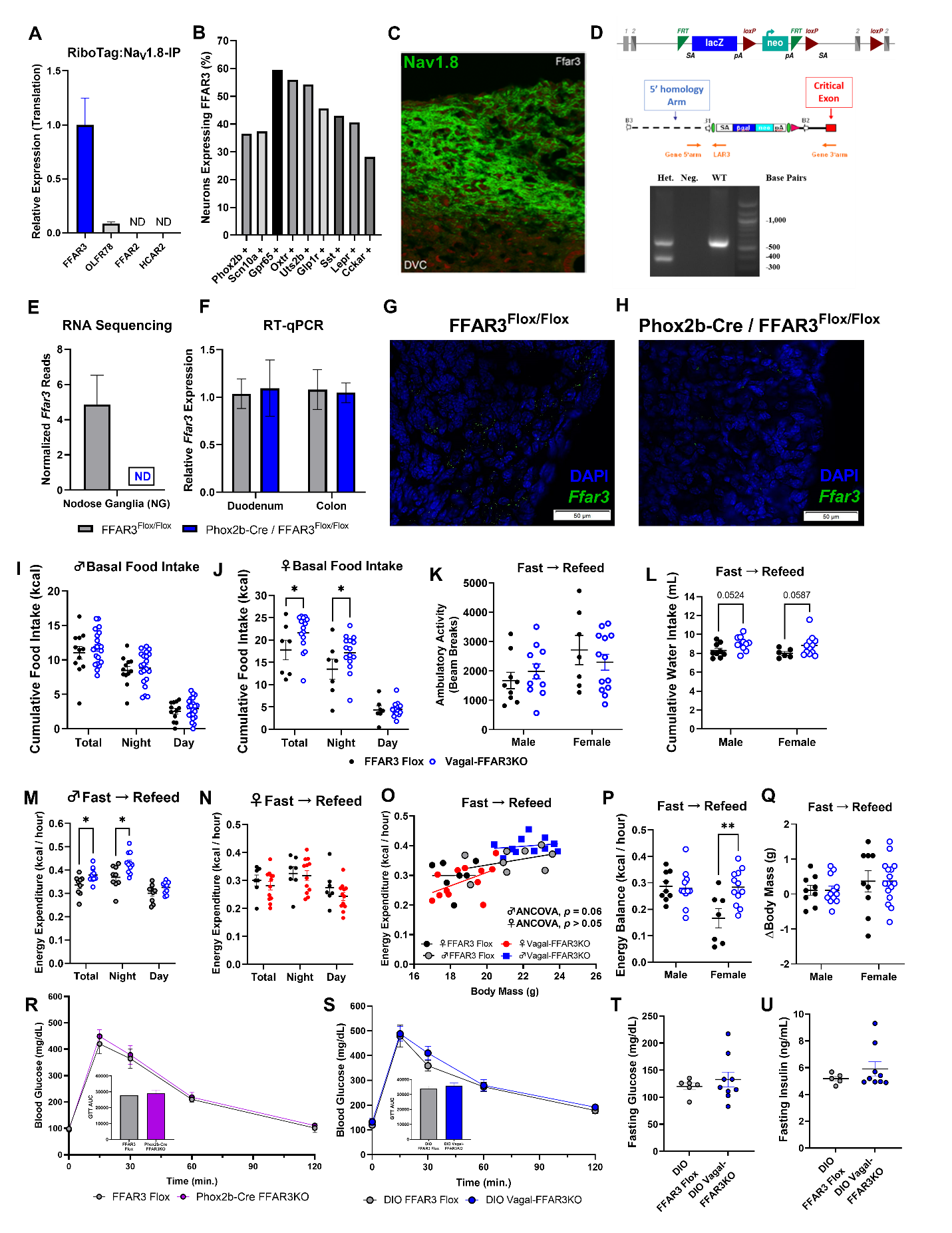


Supplemental Figure 1

(A) Immunoprecipitation and RT-qPCR of translating mRNA from the nodose ganglia of adult Nav1.8-Cre: RiboTag mice. Gene expression normalized to β-actin.

(B) Percentage of neurons expressing *Ffar3* among distinct clusters of vagal sensory neurons from target single-cell (Target-scSeq) RNA sequencing (Bai *et al.* 2019).

(C) Chromogenic *in situ* hybridization (CISH) staining of the dorsal vagal complex (DVC) demonstrating the absence of *Ffar3* mRNA.

(D) Scheme and genotyping gel for the generation of Ffar3 floxed allele generated by Dr. Brian Layden (UIC).

(E–F) RNA sequencing (E) and RT-qPCR (F) of *Ffar3* expression in nodose ganglia and intestines of FFAR3 flox and vagal-FFAR3 knockout mice.

(G–H) Fluorescent *in situ* hybridization of nodose ganglia from FFAR3 flox mice (G) and vagal-FFAR3 knockout mice (H).

(I–J) Basal food intake of FFAR3 flox and vagal-FFAR3KO littermates (*n*=12–20 males/group, 7–14 females/group).

(K–Q) Ambulatory activity (K), water consumption (L), energy expenditure (M–O), and average energy balance (P) during refeeding. Subsequent change in body mass (Q) after refeeding (*n*=8–12 females, *n*=9–11 males/group).

(R) GTT trace and area under curve of lean female FFAR3 flox and vagal-FFAR3KO mice (*n*=4–10 female mice/group, Student’s *t*-test).

(S–U) Glucose tolerance test trace and area under the curve (S), fasting glucose (T), and fasting insulin levels (U) of DIO FFAR3 flox and vagal-FFAR3KO mice (*n*=6–9 male mice/group, Student’s *t*-test).

Error bars indicate mean±SEM; two-way ANOVA with repeated measures; Fischer’s LSD **p* <0.05.


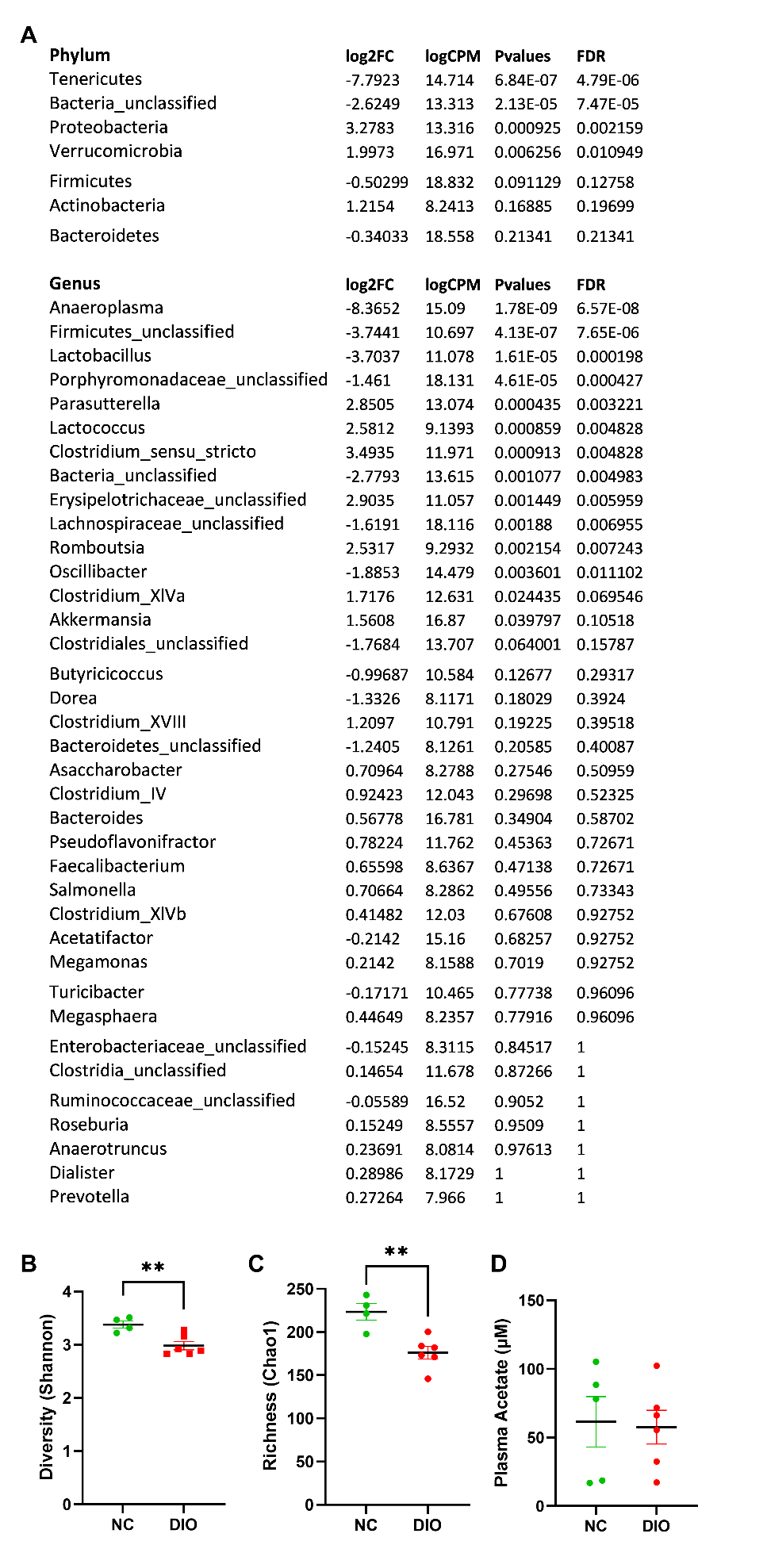


Supplemental Figure 2

(A–C) 16S sequencing results and analysis using MicrobiomeAnalyst (Chong et al., 2020). Phylum and genus abundance table expressed in log fold change (log2FC) in NC-fed vs. DIO (A), Shannon diversity (B), and chao1 richness (C) indices (*n*=4–6 mice/group).

(D) Plasma acetate levels of NC and DIO mice (Student’s *t*-test, *n*=5–6 mice/group).

Error bars indicate mean±SEM


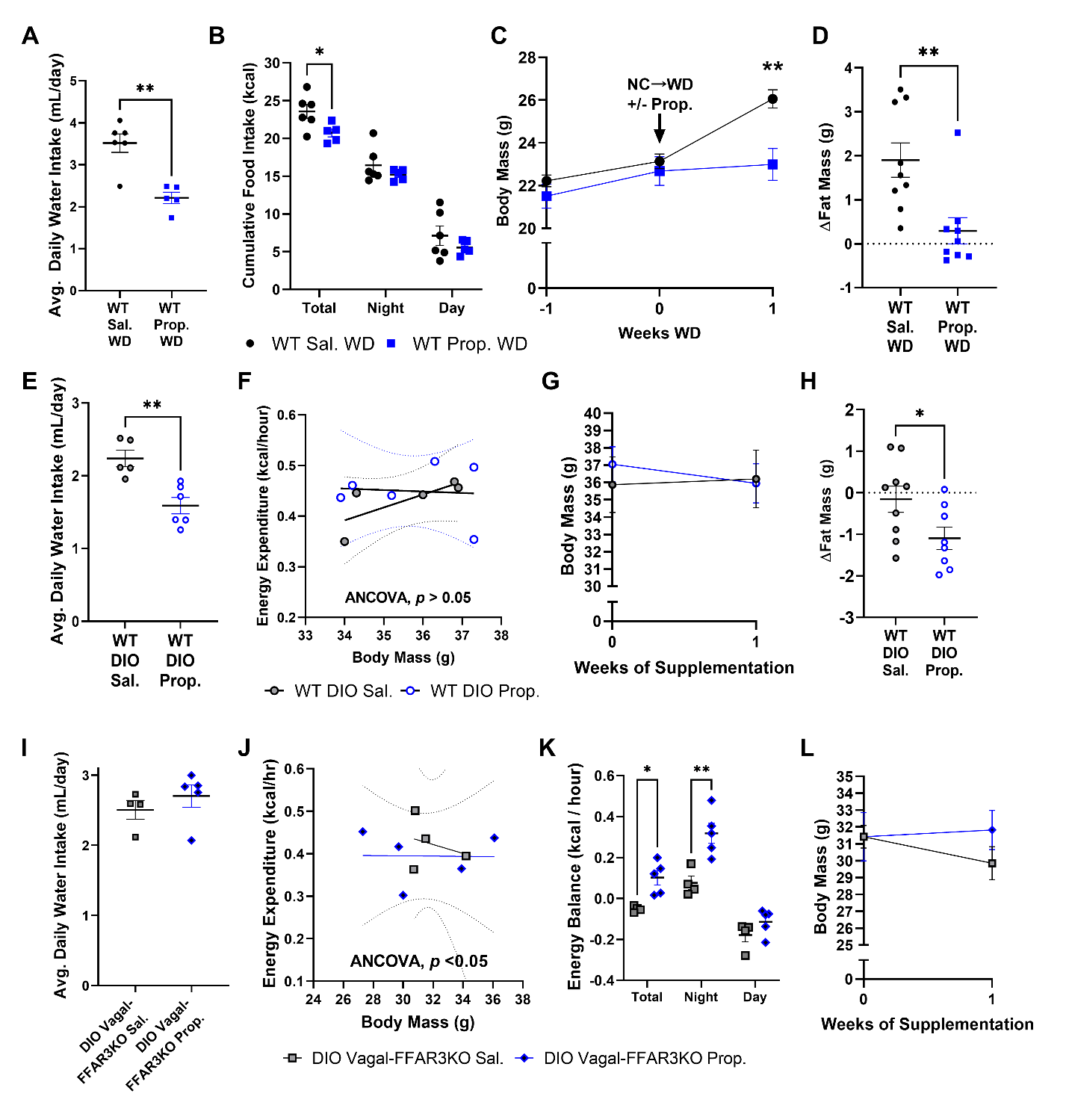


Supplemental Figure 3

(A-D) Lean wild-type (WT) mice after switch to WD-feeding with or without 25mg/mL sodium propionate in the drinking water. Daily water intake (A) and cumulative food intake (B) during first 48 hours of switch to WD with or without propionate in water (n=5-6 male mice / group; Two-Way ANOVA with repeated measures, Fischer’s LSD **p* <0.05; Student’s T-test ***p* <0.01). Longitudinal body mass over 2 weeks (C) and delta fat mass after the first week of WD-feeding (D) with or without propionate (n=8-9 mice / group, Student’s T-test ***p* <0.01).

(E-H) Diet-induced obese WT mice continued WD-feeding with 25mg/mL sodium propionate or equimolar saline administered *ad libitum* through drinking water. Average daily water intake (E), and energy expenditure vs. body mass (F) after drinking water was supplemented with propionate or saline (n=5-6 male mice / group, Two-Way ANOVA with repeated measures, Fisher’s LSD **p* <0.05, Student’s T-test ***p* <0.01). Body mass (G) and change in fat mass (H) after one week of propionate or saline drinking (n=8-9 mice / group, Student’s T-test **p* <0.05 ***p*<0.01).

(I-L) Indirect calorimetry assessment of DIO vagal-FFAR3KO mice supplemented with 25mg/mL sodium propionate in drinking water or equimolar saline. Average daily water intake (I), energy expenditure vs body mass (J), and average energy balance (K) during propionate or saline supplementation (n=4-5 mice / group, Two-Way ANOVA with repeated measures, Fischer’s LSD **p* <0.05 ***p* <0.01). Body mass (L) before and after one week of propionate or saline supplementation (n=4-5 mice / group, Student’s T-test **p* <0.05.

Error bars indicate mean ±SEM.


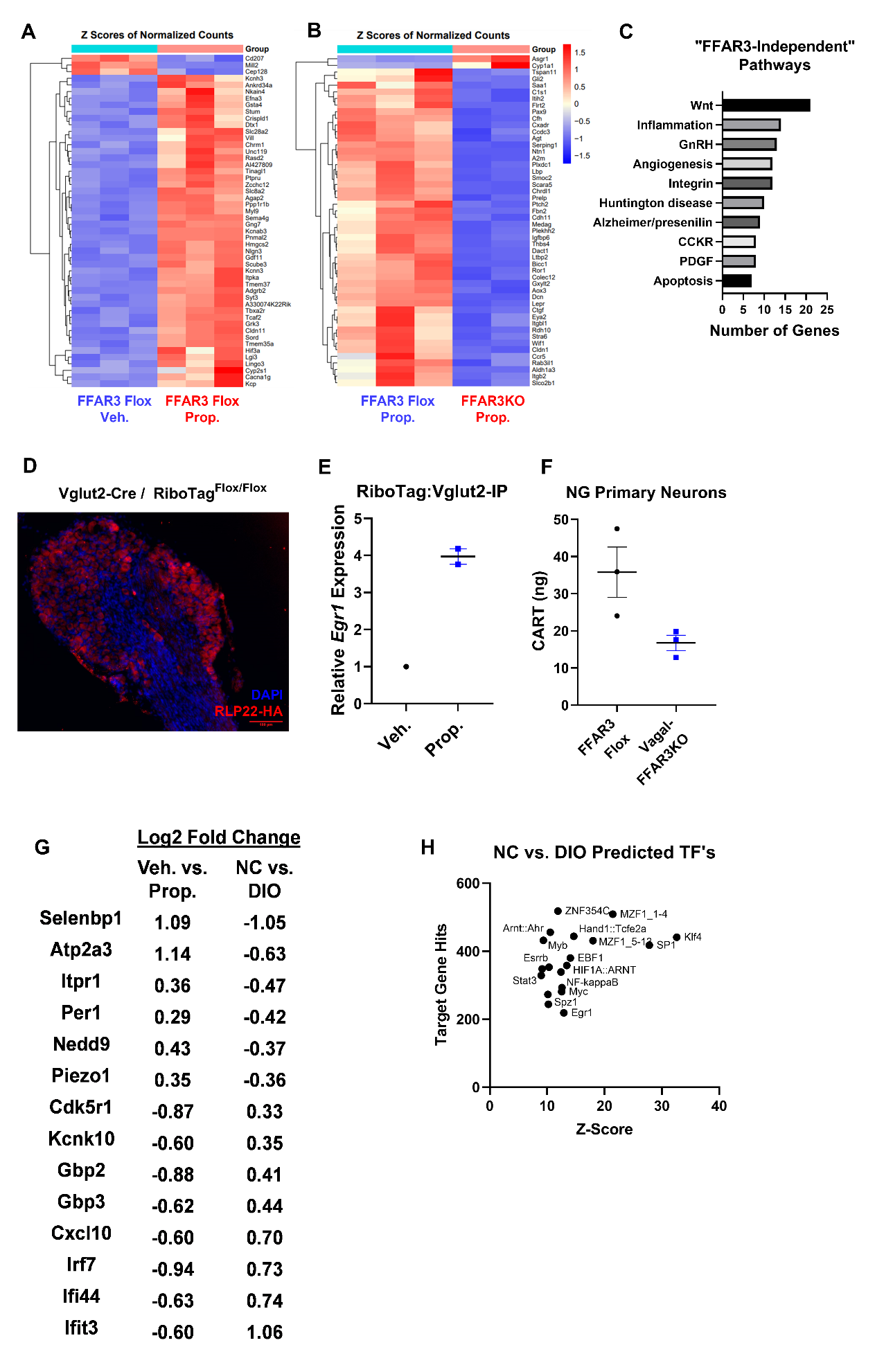


Supplemental Figure 4

(A-B) Top 50 genes altered between FFAR3 Flox vehicle vs. Propionate (A), and FFAR3 Flox Propionate vs. vagal-FFAR3KO Propionate (B).

(C) Pathway analysis of transcripts altered after propionate treatment, regardless of FFAR3 expression or deletion (see Figure 4B).

(D-E) Acute propionate treatment on nodose ganglion explants (NG) from Vglut2-Cre: RiboTag. Anti-HA immunostaining of NG demonstrating HA-tagged Rlp22 (D). RT-qPCR of immunoprecipitated NG transcripts after 2-hour treatment of vehicle or 1mM propionate (D).

(F) NG primary cultures isolated from FFAR3 flox and vagal-FFAR3KO. Primary neurons were serum starved overnight and supernatant was removed to measure secreted CART via ELISA (n=1 experimental replicate, 3 wells).

(G) Select list of genes overlapping from “FFAR3-dependent” and NC vs WD groups (n=3 replicates / group, FDR-adjusted p<0.05).

(H) Predicted transcription factors based of transcripts altered in nodose ganglia of NC vs. WD DIO mice (n=3 mice / group).

References

Bai, L., Mesgarzadeh, S., Ramesh, K.S., Huey, E.L., Liu, Y., Gray, L.A., Aitken, T.J., Chen, Y., Beutler, L.R., Ahn, J.S., et al. (2019). Genetic Identification of Vagal Sensory Neurons That Control Feeding. Cell *179*, 1129-1143.e1123. 10.1016/j.cell.2019.10.031.

Chong, J., Liu, P., Zhou, G., and Xia, J. (2020). Using MicrobiomeAnalyst for comprehensive statistical, functional, and meta-analysis of microbiome data. Nat Protoc *15*, 799-821. 10.1038/s41596-019-0264-1.

Ho Sui, S.J., Mortimer, J.R., Arenillas, D.J., Brumm, J., Walsh, C.J., Kennedy, B.P., and Wasserman, W.W. (2005). oPOSSUM: identification of over-represented transcription factor binding sites in co-expressed genes. Nucleic Acids Res *33*, 3154-3164. 10.1093/nar/gki624.
